# Integrated meta-analysis and exploratory small-sample machine learning to evaluate curcumin against osteoporosis: a preclinical evidence-based study

**DOI:** 10.3389/fendo.2026.1893878

**Published:** 2026-08-12

**Authors:** Yuzhuo Ma, Jiaojiao Bai, Rui Wang, Yijin Wang, Hongru Wei, Ying Zhang, Xuefei He, Ni Zhang

**Affiliations:** 1 Department of Nursing, Honghui Hospital, Xi’an Jiaotong University, Xi’an, Shaanxi, China; 2 Department of Critical Care Medicine, Honghui Hospital, Xi’an Jiaotong University, Xi’an, Shaanxi, China; 3 The Clinical Medical College, Chengdu University of Chinese Traditional Medicine, Chengdu, Sichuan, China; 4 Gongxian Traditional Chinese Medicine Hospital, Yibin, Sichuan, China

**Keywords:** curcumin, machine learning, meta-analysis, osteoporosis, preclinical evidence

## Abstract

**Purpose:**

To systematically evaluate the anti-osteoporotic efficacy of curcumin in preclinical models and utilize an exploratory small-sample machine learning (ML) framework to examine potential contributors to heterogeneity and generate preliminary dose–duration hypotheses.

**Methods:**

Following PRISMA guidelines, we searched eight databases for controlled *in vivo* animal studies in validated osteoporosis models that compared curcumin monotherapy with saline or vehicle controls and reported extractable bone-related outcomes, including bone mineral density (BMD) and trabecular microarchitecture. Methodological quality was assessed using the SYRCLE tool. In addition to random-effects meta-analysis, an exploratory ML framework incorporating SHapley Additive exPlanations (SHAP) and Gaussian Process Regression (GPR) was employed to examine associations between nine study-level features and effect estimates and to explore potential non-linear dose–response patterns after normalization to the Human Equivalent Dose (HED).

**Results:**

Twenty-three studies were included. Meta-analysis revealed that curcumin significantly elevated femoral BMD [standardized mean difference (SMD) = 2.73, P < 0.001], preserved trabecular microarchitecture, and enhanced biomechanical strength. Curcumin was also associated with changes in bone-remodeling and oxidative stress-related markers. Based on only 23 study-level observations, exploratory ML suggested that body weight had the largest model-dependent contribution (mean |SHAP| = 0.201). GPR modeling suggested a bell-shaped dose-response relationship, with the fitted response reaching a local maximum at an HED of approximately 32 mg/(kg·d) and an intervention duration of 8–12 weeks. These model-derived findings should not be interpreted as validated optimal dose or duration estimates.

**Conclusion:**

Curcumin exerts potent, multi-target osteoprotective effects that are associated with improved bone remodeling and oxidative stress-related indices. The exploratory integration of ML with meta-analysis suggested that biological characteristics and dosage may contribute to variability in treatment effects. However, because the ML component was constrained by the limited number of study-level observations, these model-derived findings should be regarded as hypothesis-generating signals and should not be interpreted as independently validated predictors or clinical dosing recommendations.

**Systematic review registration:**

https://www.crd.york.ac.uk/prospero/, identifier CRD420251269951.

## Introduction

Osteoporosis (OP) is a systemic metabolic bone disorder characterized by reduced bone mass and the microarchitectural deterioration of bone tissue. Its fundamental pathogenesis is rooted in a homeostatic imbalance between osteoclast-mediated bone resorption and osteoblast-mediated bone formation. This disruption progressively enhances bone fragility, thereby significantly elevating the risk of fractures (1, 2). Driven by the accelerating global demographic shift toward an aging population, OP has emerged as a formidable public health challenge. Statistics indicate that resultant fragility fractures not only lead to high morbidity and mortality but also impose a substantial economic burden on global healthcare systems (3, 4). Currently, first-line pharmacological interventions for OP primarily comprise bisphosphonates, hormone replacement therapies, and RANKL inhibitors. However, the long-term administration of these agents is frequently associated with adverse effects, including osteonecrosis of the jaw, atypical femoral fractures, and potential cardiovascular events, which substantially limit patient compliance (5, 6). Consequently, identifying candidate molecules from natural products that exhibit multi-target efficacy and low toxicity to effectively modulate bone metabolism has become a pivotal focus of contemporary orthopedic drug discovery (7, 8).

Curcumin, a natural polyphenol derived from the rhizomes of *Curcuma longa*, has attracted considerable interest due to its potent anti-inflammatory, antioxidant, and multi-target regulatory effects on bone metabolism (9, 10). Extensive preclinical evidence suggests that curcumin holds significant promise for elevating bone mineral density (BMD) and preserving bone microarchitecture across various OP models, including ovariectomized (OVX) and glucocorticoid-induced (GIOP) models (10–12). Its underlying molecular mechanisms have been proposed to involve the RANKL/OPG axis, Wnt/β-catenin signaling, and Nrf2-mediated oxidative stress regulation (13, 14). However, constrained by its poor bioavailability, existing preclinical studies exhibit marked variability in terms of dosage (10–500 mg/kg), intervention duration, and pharmacological formulations. This lack of standardization has resulted in high heterogeneity across efficacy evaluations, with divergent findings reported for key therapeutic indicators (15). Such discrepancies represent a significant barrier to the clinical translation of curcumin from bench to bedside.

To address these gaps, the present study employs a systematic meta-analysis to synthesize global preclinical evidence, providing a robust and objective assessment of curcumin’s anti-osteoporotic efficacy. While meta-analysis remains a principal method for synthesizing preclinical evidence, conventional subgroup analyses may provide limited insight into non-linear associations among continuous study-level variables, such as animal age, body weight, dosage, and intervention duration (16). We therefore incorporated machine learning (ML) as a complementary analytical approach to explore potential multidimensional patterns that may not be fully captured by traditional methods (17, 18). Given that the ML dataset was derived from a limited number of included studies, this component was primarily intended for exploratory analysis and hypothesis generation rather than for establishing definitive predictive models or clinical recommendations. Accordingly, the ML findings were interpreted alongside the conventional meta-analytic results, with careful consideration of the dataset size and study heterogeneity (19).

## Methods

This systematic review and meta-analysis was conducted in strict accordance with the Preferred Reporting Items for Systematic Reviews and Meta-Analyses (PRISMA) statement (20). Furthermore, the study protocol was prospectively registered with the International Prospective Register of Systematic Reviews (PROSPERO; registration number: CRD420251269951).

### Literature search and selection strategy

A comprehensive search was performed across eight electronic databases, comprising Web of Science, PubMed, Embase, Scopus, the Foreign Medical Literature Retrieval Service (FMRS), China National Knowledge Infrastructure (CNKI), Wanfang Data, and the VIP database. The literature retrieval process was conducted independently by two investigators to ensure objectivity and minimize bias. The search strategy utilized a combination of Medical Subject Headings (MeSH) terms and relevant free-text keywords. These term combinations (detailed in Appendix 1) were applied to identify relevant studies from database inception through December 20, 2025.

### Inclusion and exclusion criteria

This meta-analysis focused on controlled *in vivo* animal studies to systematically evaluate the comparative efficacy of curcumin against saline or placebo (vehicle control) in animal models of OP. Studies were eligible for inclusion if they met the following PICOS-based criteria: a) Population: Validated animal models of OP (e.g., OVX or GIOP); b) Study type: Original *in vivo* experimental studies; c) Outcomes: Clearly defined parameters with extractable quantitative data (e.g., BMD, trabecular microarchitecture); d) Design: Controlled trials utilizing a randomized allocation methodology. The exclusion criteria were defined as follows: a) models involving irrelevant comorbidities or other confounding metabolic bone disorders; b) *in vitro* studies; c) studies involving combination therapies or complex herbal formulas in which the independent effect of curcumin could not be isolated; d) redundant publications or studies utilizing overlapping datasets; and e) non-original research, including conference abstracts, reviews, editorials, or letters to the editor. For multi-arm studies that included both curcumin monotherapy and combined-intervention groups, only the curcumin monotherapy arm and the corresponding control arm were extracted.

### Data extraction

Following the removal of duplicate records using EndNote, two investigators (Y.M. and J.B.) independently screened titles and abstracts manually in a blinded manner to exclude studies that failed to meet the eligibility criteria. Subsequently, the full texts of potentially eligible studies were retrieved and reviewed manually to confirm their final inclusion. Any discrepancies during the selection process were resolved by consensus or through arbitration by a third reviewer (R.W.). Data extraction was performed independently and in duplicate by two researchers (Y.M. and J.B.) to ensure accuracy and minimize bias. The extracted data items included: first author and year of publication; model induction methodology; animal characteristics (body weight and age); sample size per group; details of interventions and administration routes; and treatment duration. Furthermore, primary and secondary outcomes were extracted as the mean and standard deviation (SD). For outcomes presented graphically, numerical values were digitized and extracted using GetData Graph Digitizer (Version 2.26).

### Risk of bias assessment

The risk of bias in the included studies was independently assessed by two reviewers (Y.M. and J.B.) using the SYRCLE’s Risk of Bias tool for animal studies (21). The assessment comprised ten items across six domains, specifically addressing selection, performance, detection, attrition, reporting, and other potential sources of bias. Each item was categorized as having a ‘low’, ‘high’, or ‘unclear’ risk of bias based on the degree to which the study fulfilled the predefined criteria. Any discrepancies between the reviewers during the assessment process were resolved through consensus-based discussion or arbitration by a third reviewer (R.W.) to ensure the accuracy and consistency of the findings.

### Outcome measure

The primary outcome of this study was femoral BMD. Secondary outcomes encompassed bone histomorphometry parameters, including trabecular number (Tb.N), trabecular thickness (Tb.Th), trabecular separation (Tb.Sp), and bone volume fraction (BV/TV); bone biomechanical properties, such as elastic modulus, maximum load, and structure model index (SMI); bone turnover markers, including tartrate-resistant acid phosphatase (TRACP), C-terminal telopeptide of type I collagen (CTX), procollagen type I N-propeptide (P1NP), alkaline phosphatase (ALP), osteocalcin (OC), serum calcium (Ca), and phosphorus (P); and biochemical modulators, specifically cytokines (IL-6, OPG, RANKL, and TNF-α) and oxidative stress markers (SOD, MDA, and GSH).

### Machine learning framework and interpretability analysis

To interrogate the observed heterogeneity and explore potential non-linear response patterns of curcumin’s anti-osteoporotic effects, we developed an ML framework integrating interpretable feature attribution and probabilistic modeling. The rationale for incorporating ML was to complement conventional subgroup analyses, which generally require continuous study-level variables to be categorized and may therefore obscure potentially non-linear patterns. Initially, a feature engineering process was employed to extract nine core variables from the included studies, encompassing biological characteristics (species, sex, age, and weight), experimental parameters (induction method and vehicle control), and intervention strategies (dosage, administration route, and duration). The ML dataset was constructed from 23 study-level observations. The nine candidate features were prespecified according to biological plausibility, experimental relevance, and availability across the included studies; no data-driven feature elimination was performed before model fitting. Before analysis, continuous variables were standardized, and categorical variables were encoded using predefined numerical codes. Hyperparameters for the Decision Tree and k-nearest neighbor models were tuned using restricted grid-search procedures, whereas the Gaussian Process Regression (GPR) kernel parameters were optimized by maximizing the log-marginal likelihood. To enhance clinical relevance, all dosages were normalized to the Human Equivalent Dose (HED) based on Body Surface Area (BSA) scaling (22, 23). To ensure model transparency, feature importance was initially screened using a Decision Tree (DT) algorithm. Subsequently, SHapley Additive exPlanations (SHAP) based on a k-nearest neighbor (kNN) architecture was implemented to quantify the contribution of each variable to the model-predicted standardized mean difference (SMD), which was reported as the mean absolute SHAP value. SHAP values were interpreted as measures of model-dependent feature contribution rather than evidence of independent or causal effects. A GPR model, chosen for its proficiency in non-parametric fitting and noise handling, was utilized to construct dose–response and time–response curves, with 95% confidence intervals calculated to reflect predictive uncertainty. Furthermore, correlation matrix heatmaps were generated to evaluate potential collinearity, including the correlation between species and body weight, and to describe associations between the evaluated features and the primary outcome (24, 25). Finally, a 3D dose–time–effect heatmap was synthesized to explore the interaction between dosage and intervention duration and to identify a candidate model-derived response region for curcumin’s bone-protective effects. Because of the limited size and diversity of the available dataset, particularly the small number of study-level observations relative to the nine candidate features, the ML analyses were considered susceptible to overfitting, collinearity, and instability of feature rankings and fitted response curves. The resulting patterns were therefore regarded as exploratory estimates requiring confirmation in larger and independently assembled datasets. Internal validation was performed using leave-one-study-out cross-validation. Model performance was assessed using mean absolute error (MAE), root mean squared error (RMSE), and coefficient of determination (R²). To reduce overfitting, we used prespecified features, restricted model complexity, constrained hyperparameter tuning, internal cross-validation, and explicit noise modeling in GPR.

### Statistical method

Statistical analysis and data visualization were performed using Stata SE (version 18) and Review Manager (version 5.4.0). Inter-study heterogeneity was quantitatively assessed using the I^2^ statistic. A fixed-effects model was employed if I^2^< 50%; otherwise, a random-effects model was used to provide more conservative estimates in the presence of significant heterogeneity (I^2^≥50%). For the primary outcome, femoral BMD, a 95% prediction interval was calculated under the random-effects model to reflect the expected range of effects in future comparable studies. To further explore the potential sources of heterogeneity, subgroup analyses and sensitivity analyses were systematically conducted. For femoral BMD, exploratory univariate random-effects meta-regression was additionally performed using prespecified study-level covariates, including species, OP model, administration route, vehicle control, intervention duration, age, body weight, and HED. Multivariable meta-regression was not performed because of the limited number of included studies and the sparse distribution of several covariate categories. Specifically, the leave-one-out approach was utilized for sensitivity analysis to evaluate the robustness and stability of the cumulative results. For continuous outcomes, effect sizes were expressed as standardized mean differences (SMDs) with corresponding 95% confidence intervals (CIs). For the primary outcome, femoral BMD, potential publication bias and small-study effects were assessed using a funnel plot, Egger’s regression test, and Begg’s rank correlation test. Statistical significance for all analyses was set at p < 0.05.

## Results

### Search results

**Figure 1:**
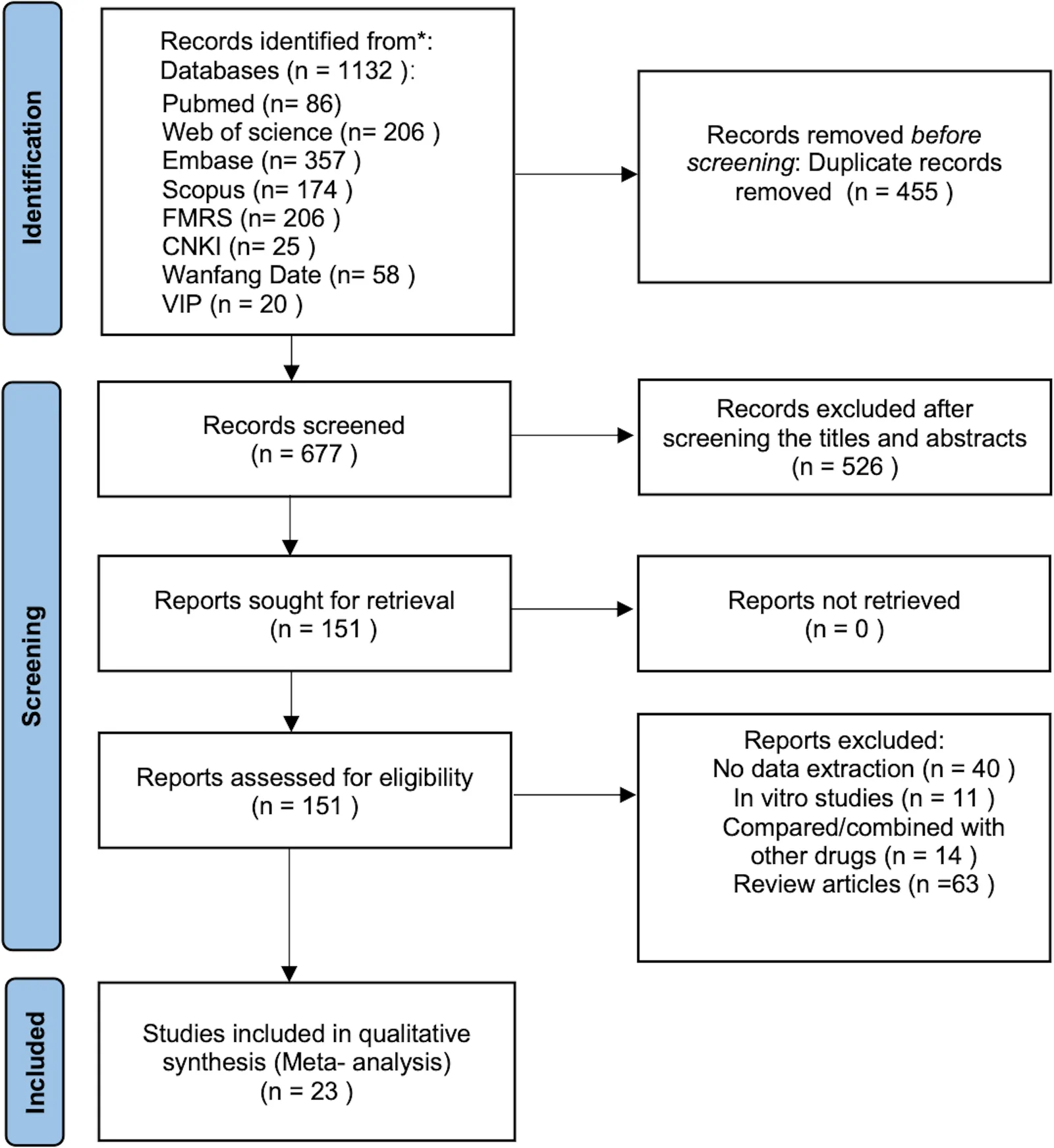
PRISMA flow chart of study selection.

The study selection process is illustrated in the PRISMA flow diagram (**Figure 1**). A total of 1,132 records were initially identified across eight electronic databases, of which 455 duplicates were removed. After screening the titles and abstracts of the remaining records, 526 irrelevant studies were excluded based on our predefined criteria. Of the 151 articles retrieved for full-text evaluation, 128 were excluded for the following reasons: (i) lack of extractable data for primary outcomes (n = 40); (ii) use of combination therapies or comparisons with alternative pharmacological agents from which the independent effect of curcumin could not be isolated (n = 14); (iii) *in vitro* study design (n = 11); and (iv) non-original research, such as reviews or editorials (n = 63). Consequently, 23 studies were eligible for inclusion in the final analysis, comprising 9 Chinese (14, 26–33) and 14 English publications (13, 34–46).

### Study characteristics

The baseline characteristics of the included studies are summarized in **Table 1**. The publication dates of the 23 eligible studies spanned from 2010 to 2025. Regarding the OP induction methodology, 14 studies utilized ovariectomy (OVX), three employed GIOP protocols, and three focused on metabolic-related models. Additionally, genetically modified (n = 2) and disuse-induced (n = 1) models were also represented. In terms of animal species, 13 studies were conducted on rats, while the remaining 10 employed mice as the experimental subjects. Vehicle controls primarily consisted of normal saline (n = 15), 0.5% CMC-Na (n = 6), and other specialized vehicles (n = 2). The routes of administration across the cohorts were predominantly oral gavage (n = 19), followed by intraperitoneal injection (n = 4). Curcumin dosages ranged from 165 μg/(kg·d) to 600 mg/(kg·d), with intervention durations lasting between 4 and 15 weeks.

**Table 1:** Characteristics of the included studies.

First author	Induction of osteoporosis	Species	Gender	Sample size		Intervention		Methods of administration	Duration of study
				IG	CG	IG	CG		
(Zhang et al., 2020) (26)	OVX	Wistar	Female	15	15	165 μg/(kg·d)	PS	Intragastric	12 weeks
(Zhang et al., 2025) (27)	OVX	SD	Female	10	10	20 mg/(kg·d)	PS	Intraperitoneal	8 weeks
(Dong et al., 2025) (28)	OVX	SD	Female	10	10	200 mg/(kg·d)	PS	Intragastric	12 weeks
(Jiang et al., 2019) (14)	OVX	SD	Female	10	10	110 mg/(kg·d)	0.5% CMC-Na	Intragastric	12 weeks
(Liu et al., 2017) (29)	OVX	SD	Female	10	10	200 mg/(kg·d)	0.5% CMC-Na	Intragastric	12 weeks
(Yang et al., 2020) (30)	OVX	SD	Female	8	8	20 mg/(kg·d)	0.5% CMC-Na	Intragastric	8 weeks
(Xu et al., 2024) (31)	OVX	BALB/c	Female	10	10	20 μmol/(kg·d)	PS	Intraperitoneal	8 weeks
(Li et al., 2024) (34)	DOM	C57BL/6J	Male	6	6	40 μmol/L, 200 μL, 3 times/week	PS	Intraperitoneal	8 weeks
(Liang et al., 2025) (35)	OVX	C57BL/6J	Female	5	5	200 mg/(kg·d)	PS	Intragastric	4 weeks
(Xin et al., 2015) (36)	HLU	SD	Male	12	12	40 mg/(kg·d)	Vehicle	Intragastric	6 weeks
(Chen, 2016) (37)	GIOP	SD	Female	6	6	100 mg/(kg·d)	0.5% CMC-Na	Intragastric	8 weeks
(Chenz, 2016) (37)	GIOP	SD	Female	6	6	100 mg/(kg·d)	0.5% CMC-Na	Intragastric	8 weeks
(Li, 2025) (39)	GIOP	SD	Female	8	8	100 mg/(kg·d)	PS	Intragastric	10 weeks
(Fan et al., 2022) (40)	DOM	C57BL/6J	Male	6	6	100 mg/(kg·d)	PS	Intragastric	8 weeks
(Kim, 2011) (41)	OVX	C57BL/6J	Female	12	13	9.5 mg/(kg·d)	PS	Intragastric	8 weeks
(Wang et al., 2021) (32)	HFD	C57BL/6J	Female	10	10	50 mg/(kg·d)	PS	Intragastric	6 weeks
(Yang et al., 2021) (33)	OVX	SD	Female	10	10	200 mg/(kg·d)	PS	Intragastric	10 weeks
(Wei et al., 2022) (42)	OVX	ICR	Female	6	6	100 mg/(kg·d)	0.5% CMC-Na	Intragastric	12 weeks
(Ahn et al., 2017) (43)	OVX	SD	Female	8	8	30 mg/(kg·d)	PS	Intragastric	15 weeks
(Wright et al., 2010) (44)	OVX	SD	Female	12	12	25 mg/(kg·d)	Vehicle	Intraperitoneal	4 weeks
(Yang et al., 2011) (46)	TOM	APP/PS1	Female	6	6	600 mg/(kg·d)	PS	Intragastric	12 weeks
(Ke et al., 2024) (13)	TOM	Tg-hRANKL	Male	10	10	200 mg/(kg·d)	PS	Intragastric	4 weeks
(Heo et al., 2014) (45)	OVX	C57BL/6J	Female	5	5	50 μmol/(kg·d)	PS	Intragastric	9 weeks

OVX, Ovariectomy; DOM, Diabetic Osteoporosis Model; HLU, Hindlimb Unloading; GIOP, Glucocorticoid-induced Osteoporosis; HFD, High-fat diet-induced; TOM, Transgenic Osteoporosis Model; IG, Intervention Group; CG, Control Group; PS, physiological saline; CMC-Na, Sodium Carboxymethylcellulose.

### Quality assessment of included studies

**Figure 2:**
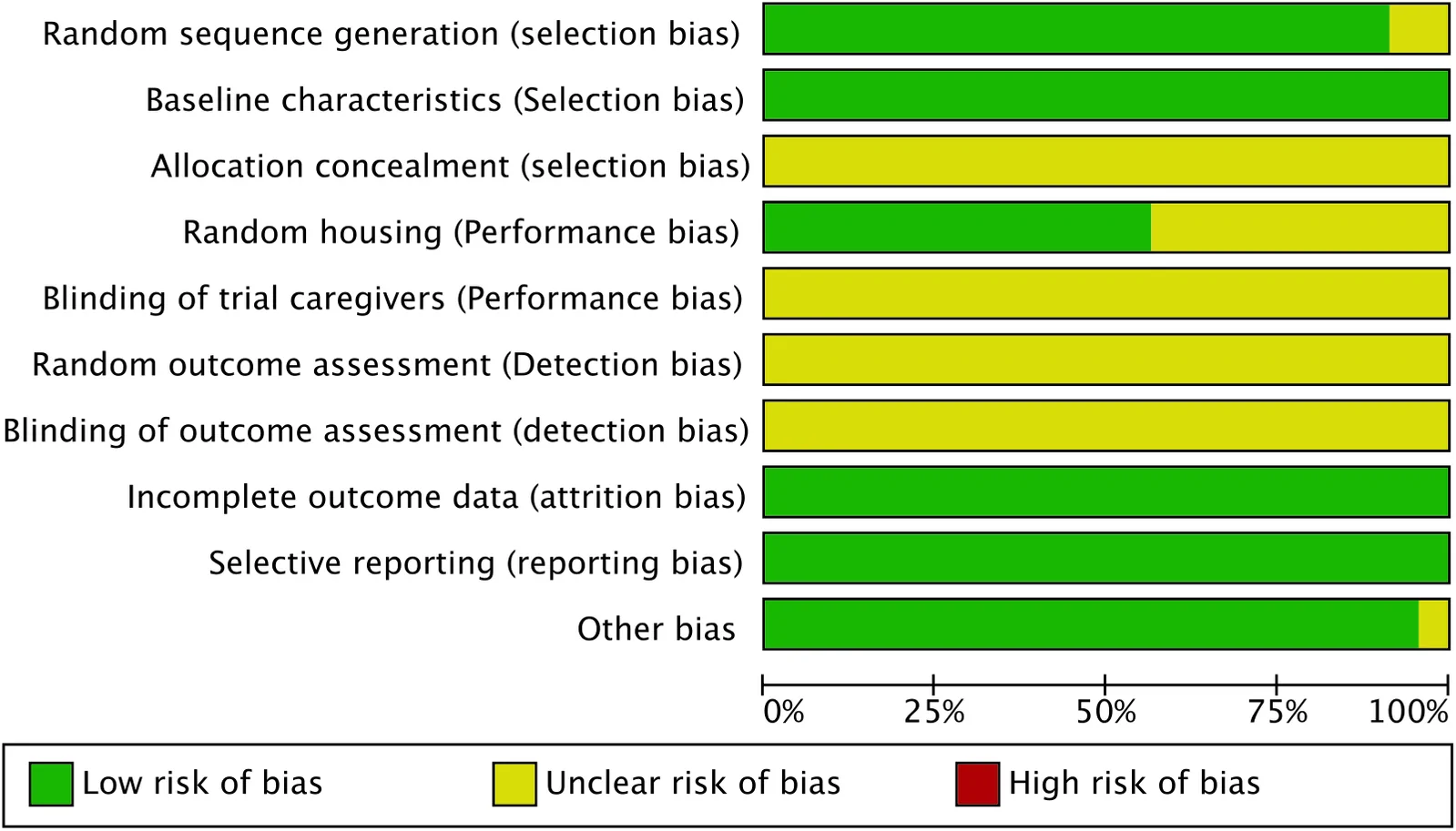
Quality of the included studies.

The risk of bias of the included studies was systematically evaluated using SYRCLE’s risk of bias tool. As illustrated in **Figure 2**, the included studies showed relatively favorable reporting in several domains, including random sequence generation, baseline characteristics, completeness of outcome data, and selective reporting. In these domains, more than 90% of the assessments were judged as having a low risk of bias, suggesting that the findings were unlikely to be substantially affected by attrition bias or selective outcome reporting. However, due to insufficient reporting in the original studies, several key domains—particularly allocation concealment, blinding of caregivers/investigators, and blinding of outcome assessment—were predominantly rated as having an unclear risk of bias. Therefore, the overall methodological quality should be interpreted with caution, especially regarding potential selection, performance, and detection biases.

### BMD and subgroup analyses

**Figure 3:**
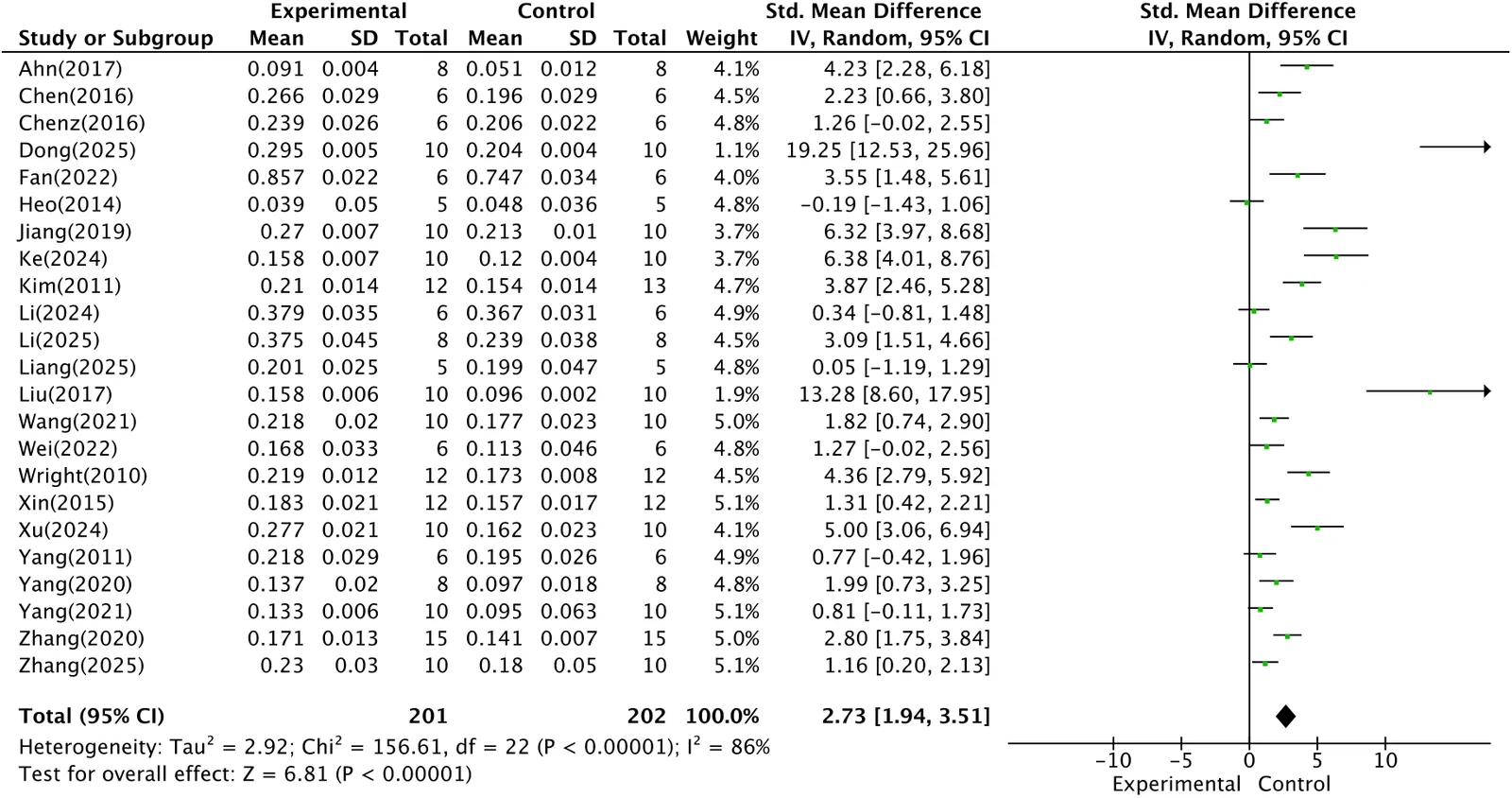
Forest plot of femoral bone mineral density (BMD) comparing the curcumin group and the control group.

This meta-analysis, synthesizing data from 23 studies, corroborated the efficacy of curcumin in enhancing BMD within animal models of OP. As illustrated in **Figure 3**, the curcumin-treated groups exhibited significantly higher femoral BMD compared to control groups (SMD = 2.73, 95% CI = 1.94-3.51, p < 0.001). The corresponding 95% prediction interval was -0.92 to 6.38, indicating substantial between-study variability. Although the pooled average effect favored curcumin, the prediction interval crossed the null value, suggesting that the magnitude of benefit may vary across different preclinical settings. To interrogate the substantial heterogeneity observed in the pooled estimates, a systematic subgroup analysis was conducted across multiple dimensions, including induction methodology, species, sex, vehicle type, dosage, and intervention duration (**Table 2**). Although the I^2^ values diminished in certain subsets (e.g., GIOP), heterogeneity remained pronounced in primary subgroups like OVX, suggesting that model-specific differences only partially account for the overall variance. Subgroup analysis further revealed that the osteoprotective effects of curcumin are highly context-dependent, peaking at dosages > 150 mg/(kg·d) and intervention durations ≥12 weeks (SMDs of 4.97 and 5.23, respectively). Notably, rat models exhibited greater sensitivity to curcumin than mouse models (SMD: 3.30 vs. 2.12). Furthermore, cohorts utilizing CMC-Na as a vehicle demonstrated superior therapeutic outcomes, highlighting the critical role of the suspension medium in modulating bioavailability. Given that conventional subgroup analysis proved insufficient to fully resolve the intricate roots of heterogeneity, exploratory univariate random-effects meta-regression was further performed for femoral BMD. As shown in **Table 3**, none of the examined covariates significantly explained the between-study heterogeneity. Body weight showed a positive but statistically non-significant trend, whereas species, OP model, administration route, vehicle control, intervention duration, age, and HED were not significantly associated with the effect size.

**Table 2:** Subgroup analysis of BMD based on multiple factors related to curcumin treatment.

Subgroup	Standardized mean difference (95% confidence interval)	I^2^(%)	p value
Model			
OVX	3.34 [2.13, 4.56]	90	0.000
GIOP	2.11 [1.05, 3.18]	36	0.000
Metabolism-related	1.71 [0.14, 3.29]	75	0.000
Other	2.53 [0.21, 4.85]	89	0.000
Species			
Rat	3.30 [2.20, 4.40]	87	0.000
Mice	2.12 [0.96, 3.28]	85	0.000
Gender			
Male	2.64 [0.59, 4.69]	88	0.000
Female	2.78 [1.89, 3.66]	86	0.000
Control Group			
PS	2.54 [1.56, 3.53]	87	0.000
CMC-Na	3.47 [1.60, 5.35]	87	0.000
Vehicle	2.76 [-0.21, 5.74]	91	0.07
Dose			
≤ 50 mg/(kg·d)	2.50 [1.58, 3.42]	82	0.000
> 50 and ≤150 mg/(kg·d)	2.74 [1.46, 4.01]	73	0.000
> 150 mg/(kg·d)	4.97 [2.22, 7.72]	93	0.000
Methods of administration			
Intragastric	2.78 [1.90, 3.66]	86	0.000
Intraperitoneal	2.60 [0.54, 4.65]	90	0.01
Duration of intervention			
≤ 6 weeks	2.56 [0.89, 4.24]	88	0.003
8-10 weeks	1.96 [1.11, 2.82]	78	0.000
≥ 12 weeks	5.23 [2.90, 7.57]	91	0.000

**Table 3:** Exploratory univariate random-effects meta-regression for femoral BMD.

Covariate	k	Coefficient	95% CI	P value
Species, rat vs mouse	23	2.77	-0.83 to 6.36	0.125
OVX model vs non-OVX	23	1.37	-2.58 to 5.31	0.480
Oral administration vs non-oral	23	1.34	-3.62 to 6.30	0.580
Saline control vs other vehicle	23	-1.56	-5.44 to 2.32	0.413
Intervention duration, per week	23	0.31	-0.34 to 0.96	0.333
Age, per month	20	0.006	-0.65 to 0.66	0.985
Body weight, per gram	16	0.015	-0.003 to 0.034	0.090
HED, per mg/(kg·d)	21	0.104	-0.050 to 0.257	0.174

### Bone histomorphometry

**Figure 4:**
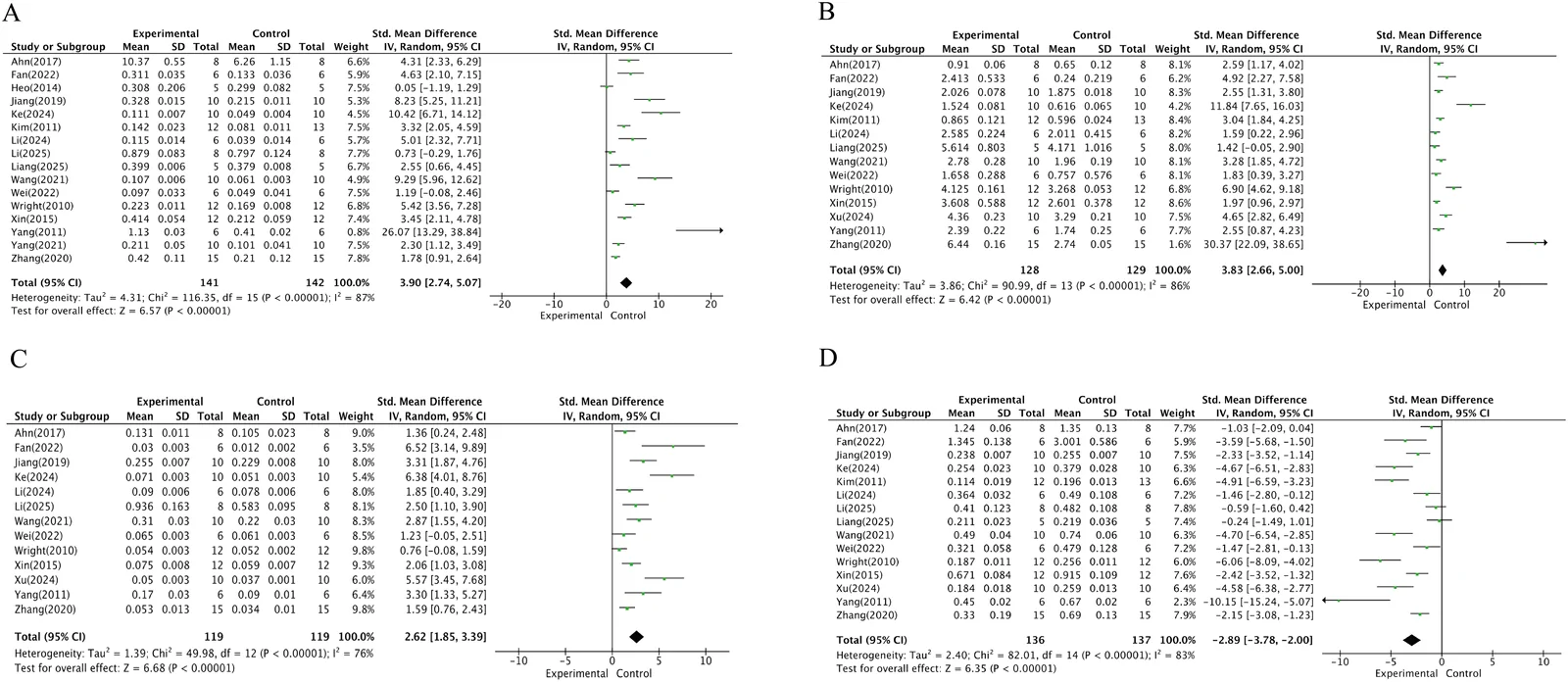
Forest plot. **(A)** BV/TV; **(B)** Tb.N; **(C)** Tb.Th; **(D)** Tb.Sp.

The restorative effects of curcumin on trabecular microarchitecture in OP models are summarized in **Figure 4**. As shown in **Figure 4**, data from 16 studies revealed a significant augmentation of BV/TV in the curcumin-treated groups (SMD = 3.90, 95% CI = 2.74-5.07, p < 0.001). Similarly, curcumin treatment led to a substantial increase in Tb.N across 14 included studies (SMD = 3.83, 95% CI = 2.66-5.00, p < 0.001; **Figure 4**). Furthermore, pooled estimates from 13 studies demonstrated that curcumin significantly enhanced Tb.Th (SMD = 2.62, 95% CI = 1.85-3.99, p < 0.001; **Figure 4**). Consistent with these improvements, a significant reduction in Tb.Sp was observed in 15 studies (SMD = -2.89, 95% CI =-3.78 to -2.00, p < 0.001; **Figure 4**).

### Bone biomechanical parameters

**Figure 5:**
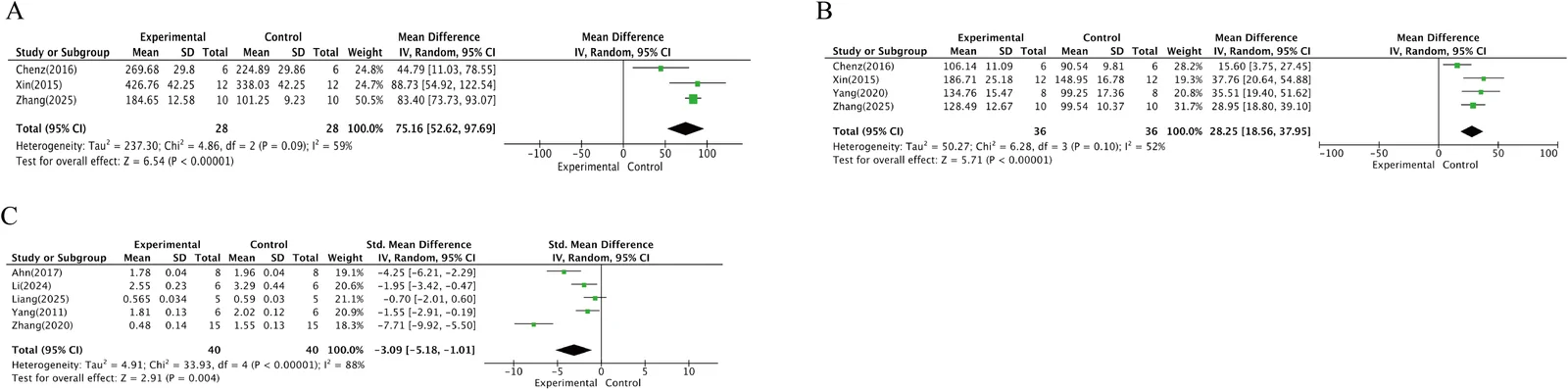
Forest plot. **(A)** Elastic modulus; **(B)** Maximum load; **(C)** Structure model index (SMI).

The impact of curcumin on the biomechanical properties of bone in OP models is presented in **Figure 5**. A pooled analysis of three studies suggested that curcumin significantly enhanced the elastic modulus (MD = 75.16, 95% CI = 52.62-97.69, p < 0.001; **Figure 5**), although this finding was based on limited evidence. Similarly, data synthesized from four studies revealed a substantial increase in maximum load capacity (MD = 28.25, 95% CI = 18.56-37.95, p < 0.001; **Figure 5**). Furthermore, five studies reported that curcumin effectively lowered SMI (SMD = -3.09, 95% CI = -5.18 to -1.01, p = 0.004; **Figure 5**).

### Biochemical markers of bone metabolism

**Figure 6:**
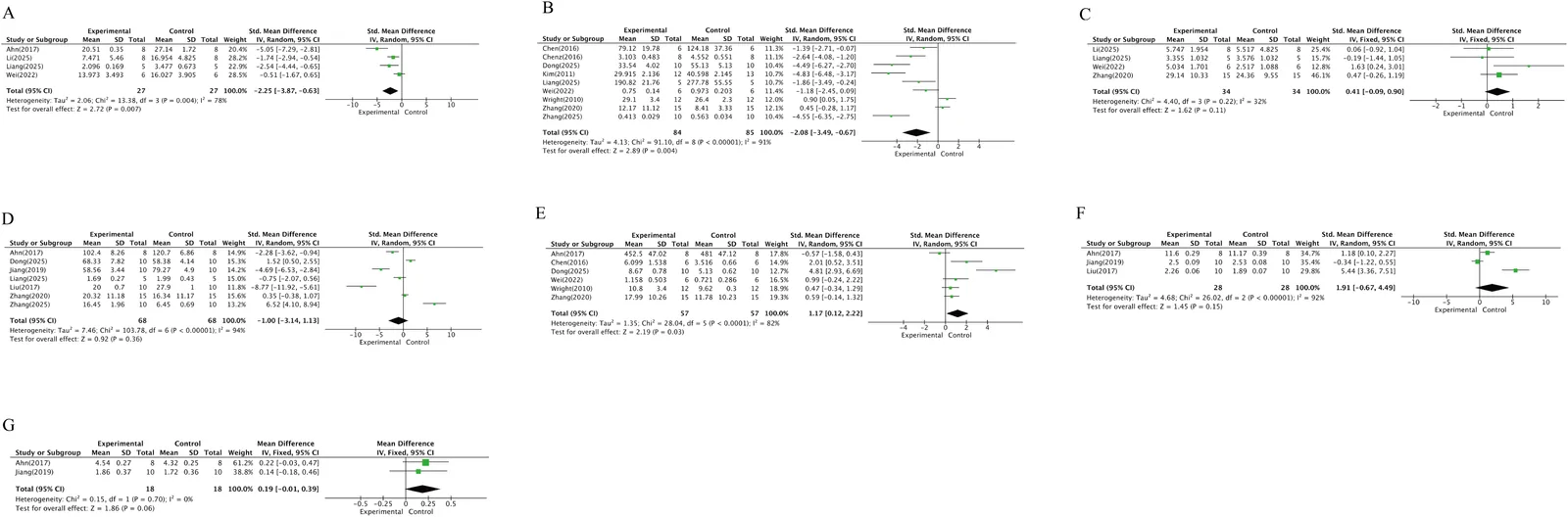
Forest plot. **(A)** TRACP; **(B)** CTX; **(C)** P1NP; **(D)** ALP; **(E)** OC; **(F)** S-Ca; **(G)** S-P.

**Figure 6** summarizes the meta-analysis results regarding the effects of curcumin on bone metabolism and biochemical markers in animal models of OP. Regarding bone resorption and turnover markers (**Figure 6A**), a pooled analysis of four studies demonstrated a significant reduction in TRACP levels (SMD = -2.25, 95% CI = -3.87 to -0.63, p = 0.007). Similarly, data synthesized from nine studies indicated that curcumin substantially lowered CTX concentrations (SMD = -2.08, 95% CI = -3.49 to -0.67, p = 0.004). In contrast, meta-analysis of four studies showed no significant impact on P1NP levels (SMD = 0.41, 95% CI = -0.09 to 0.90, p = 0.11). Concerning bone formation and mineral metabolism (**Figure 6D**), pooled estimates from seven studies revealed that curcumin did not significantly alter ALP levels (SMD = -1.00, 95% CI = -3.14 to 1.13, p = 0.36). However, six studies corroborated that curcumin significantly elevated OC levels (SMD = 1.17, 95% CI = 0.12-2.22, p = 0.03). In terms of serum mineral status, the pooled results from three studies for serum calcium (S-Ca) showed no statistical significance (SMD = 1.91, 95% CI = -0.67-4.49, p = 0.15). Similarly, curcumin intervention did not yield a significant change in serum phosphorus (S-P) levels (MD = 0.19, 95% CI = -0.01 to 0.39, p = 0.06).

### Bone metabolism pathways and pro-inflammatory cytokines

**Figure 7:**
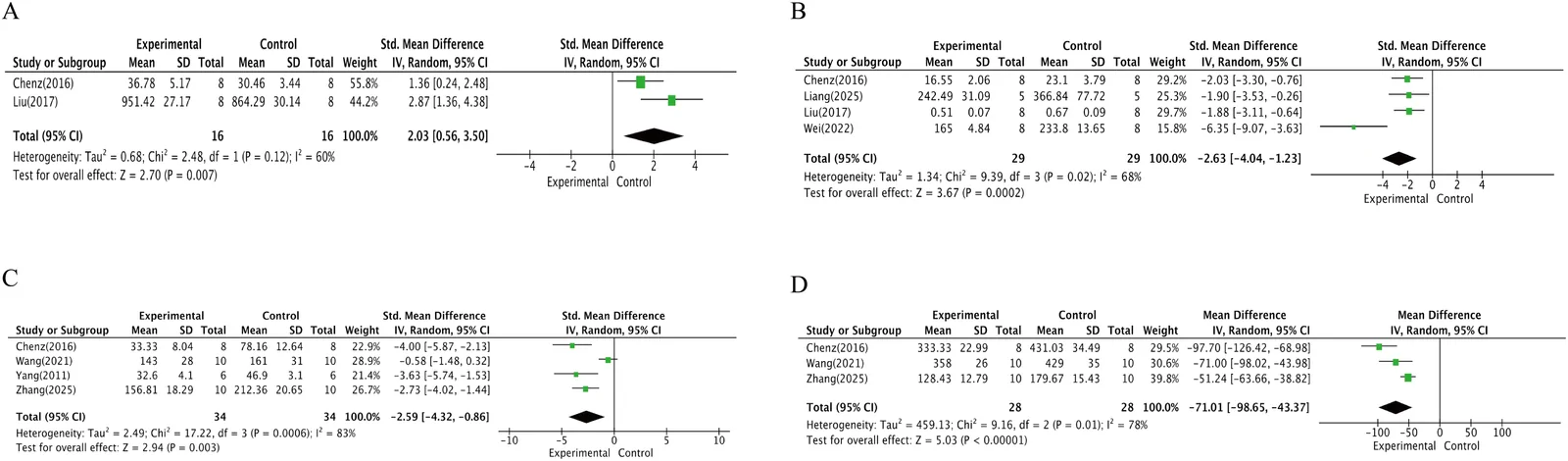
Forest plot. **(A)** OPG; **(B)** RANKL; **(C)** IL-6; **(D)** TNF-α.

**Figure 7** summarizes the pooled effects of curcumin on bone remodeling signaling pathways and inflammatory markers in animal models of OP. As illustrated in **Figure 7A**, a pooled analysis of two studies suggested that curcumin significantly upregulated OPG expression (SMD = 2.03, 95% CI = 0.56-3.50, p = 0.007), although this estimate was based on limited evidence. Correspondingly, data synthesized from four studies revealed a marked downregulation of RANKL levels (SMD = -2.63, 95% CI = -4.04 to -1.23, p < 0.001). Regarding pro-inflammatory cytokines (**Figure 7C**), the results from four studies showed that curcumin treatment led to a significant reduction in IL-6 concentrations (SMD = -2.59, 95% CI = -4.32 to -0.86, p = 0.003). Furthermore, a meta-analysis of three studies suggested that curcumin significantly suppressed TNF-α levels (MD = -71.01, 95% CI = -98.65 to -43.37, p < 0.001).

### Biomarkers of oxidative stress

**Figure 8:**
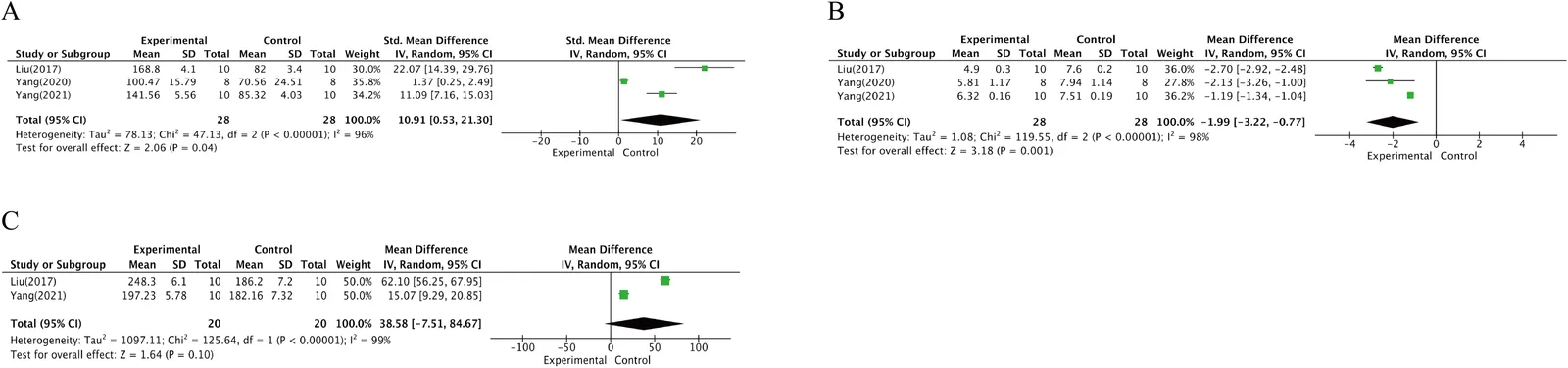
Forest plot. **(A)** SOD; **(B)** MDA; **(C)** GSH.

**Figure 8** illustrates the pooled effects of curcumin on oxidative stress markers within animal models of OP. As shown in **Figure 8**, a meta-analysis of three studies suggested that curcumin significantly enhanced the activity of the antioxidant enzyme SOD (SMD = 10.91, 95% CI = 0.53 to 21.30, p = 0.04). Furthermore, synthesized data from three studies indicated that curcumin was associated with reduced levels of MDA, a marker of lipid peroxidation (MD = -1.99, 95% CI = -3.22 to -0.77, p = 0.001; **Figure 8**). Regarding the antioxidant GSH, pooled results from two studies showed a moderate increase, although this trend did not reach statistical significance (MD = 38.58, 95% CI = -7.51 to 84.67, p = 0.10; **Figure 8**).

### Sensitivity analysis

**Figure 9:**
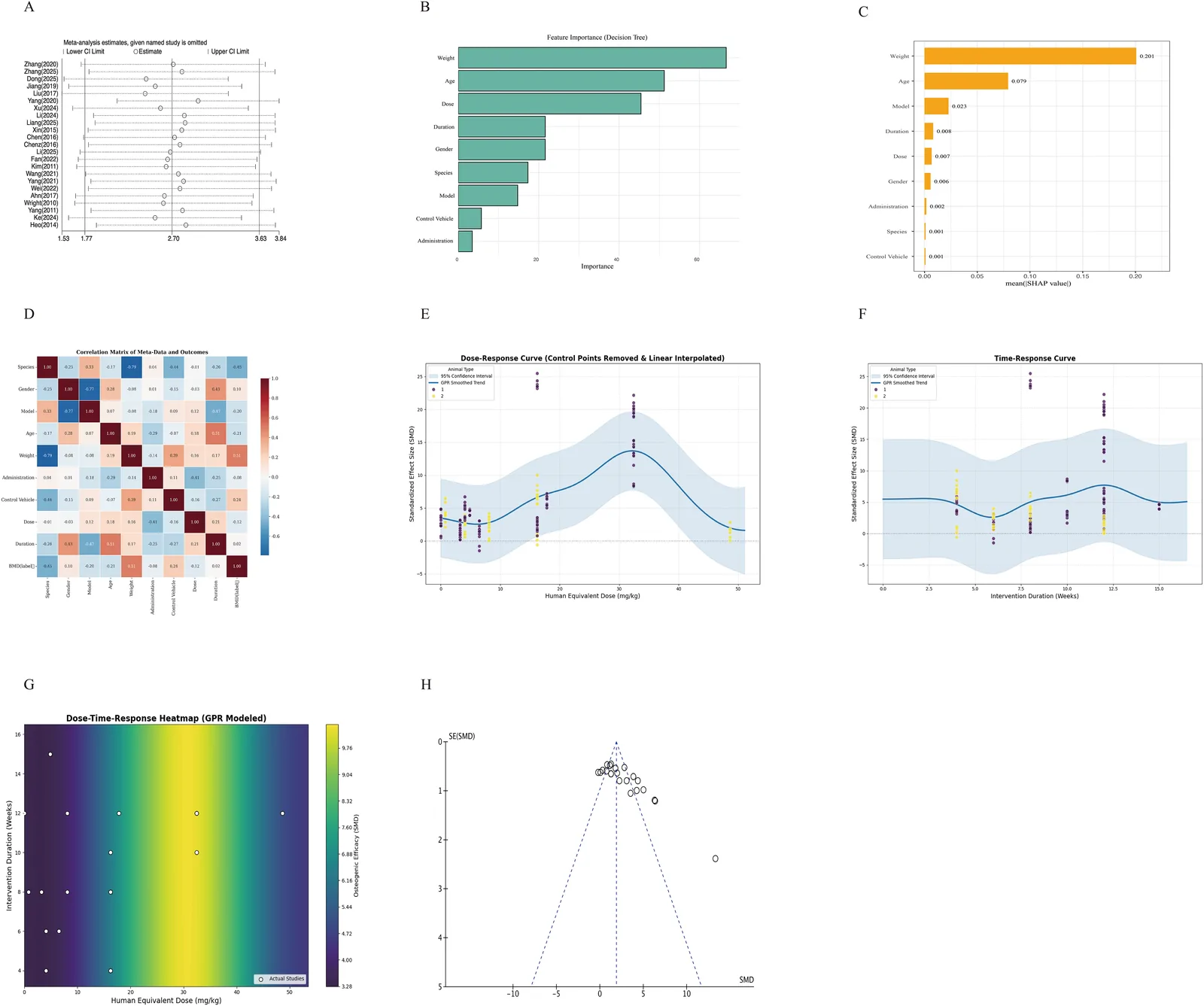
**(A)** Sensitivity analysis of femoral BMD; **(B)** decision tree for feature importance; **(C)** SHAP attribution analysis; **(D)** feature correlation matrix; **(E)** Gaussian process regression (GPR) for dose-response; **(F)** temporal-response curve; **(G)** 3D dose-time interaction heatmap. **(H)** funnel plot for femoral BMD.

To assess the robustness of the pooled estimates for the primary outcome (femoral BMD), a sensitivity analysis was performed, as illustrated in **Figure 9**. A leave-one-out approach was utilized to systematically evaluate the influence of each of the 23 included studies on the overall effect size. The results showed that the pooled effect estimates remained consistently close to the overall estimate after sequential omission of each individual study, with all recalculated confidence intervals remaining above the null effect line. These findings indicate that the overall meta-analytic estimate was stable and was not materially altered by the exclusion of any single study, suggesting that the observed osteoprotective effect of curcumin was not driven by an individual outlier.

### Machine learning analysis of core predictors and dose-response patterns

Our exploratory ML analysis examined potential study-level contributors and dynamic response patterns associated with the anti-osteoporotic efficacy of curcumin. Leave-one-study-out cross-validation indicated limited out-of-sample predictive performance. The Decision Tree model yielded an MAE of 5.057, an RMSE of 6.783, and an R² of -0.357; the kNN model yielded an MAE of 4.533, an RMSE of 6.327, and an R² of -0.181; and the GPR model yielded an MAE of 4.681, an RMSE of 7.156, and an R² of -0.510. These negative cross-validated R² values indicated that the models did not consistently outperform prediction based on the training-set mean; therefore, the ML outputs were retained for exploratory feature ranking and visualization rather than interpreted as reliable predictive models. As illustrated in **Figure 9B**, feature importance ranking using the Decision Tree and SHAP attribution assigned the largest relative model contribution to body weight, yielding an importance score exceeding 60. Subsequent SHAP analysis similarly showed that body weight had the largest mean absolute attribution within the fitted model (mean |SHAP value| = 0.201). These findings reflect the structure of the current dataset and do not establish an independent or causal effect of body weight. The correlation matrix (**Figure 9**) revealed intricate inter-variable associations, notably a strong inverse correlation between species and weight (r = -0.79). BMD exhibited a positive correlation with weight (r = 0.51). However, because body weight was strongly correlated with species, this association should be interpreted descriptively and may partly reflect differences between animal species or other correlated experimental characteristics. GPR modeling of the dose–response relationship (**Figure 9**) suggested a non-linear, bell-shaped pattern, with the model-predicted effect increasing alongside the HED before declining. The fitted curve reached its maximum at an HED of approximately 32 mg/(kg·d) (predicted SMD ≈ 13.8). Given the limited and heterogeneous study-level dataset, this value should be interpreted as an exploratory model output rather than as a precise estimate of efficacy or an optimal clinical dose. This dose range broadly overlapped with the subgroup showing the largest pooled effect; however, the absolute predicted SMD should be interpreted cautiously because of the limited number of study-level observations and the exploratory nature of the model. Concurrently, the fitted temporal-response curve (**Figure 9**) reached a local maximum (SMD ≈ 7.8) at approximately 12 weeks of continuous intervention. Exploratory evaluation of the dose–time interaction heatmap (**Figure 9**) suggested a candidate model-derived response region for bone protection, characterized by an HED of 25–40 mg/(kg·d) and an intervention duration of 8–12 weeks. Model-predicted effects were lower outside this region, particularly for intervention durations shorter than six weeks. However, this pattern requires confirmation in larger preclinical datasets and should not be interpreted as a clinically established therapeutic window.

### Publication bias assessment

Publication bias and small-study effects were evaluated for the primary outcome, femoral BMD. As shown in **Figure 9**, the funnel plot showed visual asymmetry, suggesting the possible presence of publication bias or small-study effects. Egger’s regression test indicated significant funnel-plot asymmetry (intercept = 6.89, P < 0.001), and Begg’s rank correlation test yielded a consistent result (Kendall’s τ = 0.65, P < 0.001). However, given the substantial heterogeneity across the included preclinical studies, this asymmetry should be interpreted cautiously and may also reflect differences in animal models, intervention protocols, curcumin formulations, or outcome-measurement methods.

## Discussion

The present study, through a systematic evaluation of 23 preclinical trials, corroborates the potent osteoprotective effects of curcumin across various OP models, including OVX, GIOP, and disuse-induced models. Our meta-analytical findings demonstrate that curcumin not only significantly elevates femoral BMD (SMD = 2.73) but also confers multi-faceted therapeutic benefits, characterized by the preservation of trabecular microarchitecture (BV/TV, Tb.N, and Tb.Th), enhancement of biomechanical strength (maximum load and elastic modulus), and restoration of bone metabolic homeostasis. A complementary component of this research was the exploratory integration of ML algorithms with conventional meta-analysis. This approach allowed us to examine potential multidimensional and non-linear patterns among study-level characteristics that may not be fully represented by categorical subgroup analyses. Within the fitted models, body weight and age showed relatively high feature contributions; however, these findings are conditional on the available dataset and do not establish that these characteristics independently determine therapeutic efficacy. Furthermore, the model-derived dose and duration ranges should be considered preliminary hypotheses for subsequent validation rather than precisely defined therapeutic optima.

The available preclinical evidence suggests that the osteoprotective effects of curcumin may be related to the coupling process between osteoblasts and osteoclasts within the bone remodeling cycle (25). Our meta-analysis showed that curcumin treatment was associated with changes in the RANKL/OPG axis in bone tissue. Mechanistically, previous studies have reported that curcumin may interfere with the binding of RANKL to its receptor RANK, thereby attenuating downstream signaling cascades, such as NF-κB or MAPK. This inhibition subsequently downregulates the expression of NFATc1, the master transcription factor for osteoclast differentiation, thereby attenuating osteoclastogenesis and maturation at the upstream level (47, 48). These molecular findings are further supported by our biochemical data, which show a significant reduction in TRACP activity and CTX levels—key indicators of osteoclast-mediated bone matrix degradation.

Concurrently, curcumin may exert pro-osteogenic effects partly associated with the Wnt/β-catenin signaling pathway. Previous studies have reported that curcumin may promote β-catenin nuclear translocation and osteogenic gene transcription, thereby supporting the differentiation of bone marrow mesenchymal stem cells (BMSCs) into osteoblasts (49, 50). The significant elevation of OC observed in our study serves as a robust indicator of enhanced osteoblastic mineralization and accelerated bone matrix synthesis. This bifunctional regulatory pattern—characterized by the simultaneous inhibition of resorption and promotion of formation—underpins the pharmacological efficacy of curcumin in restoring bone mass and microarchitecture across diverse pathological models.

The pathogenesis of OP is frequently characterized by a pro-inflammatory milieu and oxidative damage within the bone microenvironment, which curcumin effectively mitigates through its pleiotropic intervention capabilities (9, 10). By reducing the expression of pro-inflammatory cytokines, specifically IL-6 and TNF-α, curcumin may attenuate inflammation-induced RANKL expression. This intervention effectively interrupts the deleterious “inflammation-bone loss” cycle. Such modulation of the inflammatory microenvironment establishes a favorable biological foundation for the survival and functional integrity of osteoblasts (51, 52).

Beyond its reported radical-scavenging activity, curcumin has been suggested to support endogenous antioxidant machinery within bone cells, potentially through the Nrf2/HO-1 signaling pathway (53). In the present study, the marked elevation of SOD activity alongside the reduction in MDA levels signifies that curcumin effectively neutralizes excessive reactive oxygen species (ROS) in bone tissue. This curcumin-mediated restoration of redox homeostasis serves to arrest ROS-induced apoptotic signaling. Consequently, it preserves cellular viability and maintains the structural integrity of the bone matrix. Collectively, this synergistic anti-inflammatory and antioxidant interplay represents a cornerstone of the systemic protective network exerted by curcumin against OP (54, 55).

While traditional meta-analysis is often constrained by the limitations of subgroup analysis, it frequently struggles to interrogate the complex, non-linear interactions among continuous variables in the presence of high heterogeneity. In contrast, the integration of SHAP attribution analysis in our study suggested that body weight had the largest model-dependent contribution (SHAP value = 0.201), with an importance score higher than that of intervention dosage. The feature correlation matrix (**Figure 9**) further showed a strong inverse correlation between species and weight. This finding may partly explain the observed discrepancy in treatment sensitivity, specifically why rat models (SMD = 3.30) exhibited a greater response to curcumin than mouse models (SMD = 2.12). Our ML framework served as an exploratory complement to conventional statistical analyses, which often fail to capture the intricate non-linear relationships between baseline characteristics and therapeutic outcomes. These results suggest that biological traits may contribute to variability in the evaluation of preclinical research. Nevertheless, substantial residual heterogeneity remained after subgroup analyses, exploratory univariate meta-regression, sensitivity analysis, and ML-based feature attribution. This residual heterogeneity may reflect unmeasured or incompletely reported differences in animal strain, baseline disease severity, OP induction protocol, skeletal site assessed, curcumin formulation and bioavailability, intervention timing, measurement technique, and methodological quality. Therefore, the pooled estimates should be interpreted as average effects across heterogeneous preclinical settings rather than as a uniform magnitude of benefit expected under all experimental conditions.

The accurate translation of experimental dosages to clinical regimens remains a significant bottleneck in natural product research. Utilizing an exploratory GPR framework, our study generated a model-derived estimate around an HED of approximately 32 mg/(kg·d), at which the fitted response curve reached its maximum. This ML-derived finding was broadly consistent with the direction of our subgroup analysis, where raw dosages > 150 mg/(kg·d) yielded the most pronounced pooled effect size (SMD = 4.97). However, this consistency should not be interpreted as independent validation because both analyses were derived from the same set of included studies. Regarding the temporal profile, the predictive model exhibited a local maximum at approximately 12 weeks, suggesting a preliminary candidate intervention duration rather than a validated optimal treatment period. Furthermore, cohorts utilizing CMC-Na as a vehicle demonstrated superior pooled effects, likely due to the enhanced dispersibility and bioavailability of curcumin afforded by this medium. These insights provide hypothesis-generating evidence for future standardized preclinical experiments rather than direct guidance for clinical dosing strategies.

Several limitations of the present study warrant consideration. First, the implementation of blinding and allocation concealment was frequently under-reported in the included studies, which may introduce potential performance bias. To address this, we rigorously utilized the SYRCLE’s tool to define and categorize the risk of bias across all included literature. In addition, the relatively small sample sizes of individual animal experiments may have reduced the precision of study-specific estimates and increased susceptibility to random error. Second, funnel-plot asymmetry and significant Egger’s and Begg’s tests suggested potential publication bias or small-study effects for femoral BMD, which may have inflated the pooled estimate. However, this finding should be interpreted cautiously and should not be regarded as definitive evidence of publication bias. Third, the clinical potential of curcumin is inevitably hampered by its poor bioavailability, despite the high potency demonstrated in preclinical models. Furthermore, there is a lack of standardized evidence regarding the comparative therapeutic enhancement afforded by different pharmacological formulations, such as nanocapsules or micelles. Fourth, the ML component was based on a relatively small dataset derived from 23 included studies. The limited number of observations relative to the nine evaluated features represented a central methodological constraint of the ML component, reducing model stability and increasing the risk of overfitting. In addition, heterogeneity in animal species, OP models, vehicles, curcumin formulations, administration routes, doses, and intervention durations resulted in sparse representation of some feature combinations. Correlations among variables, particularly between species and body weight, further limited the ability to distinguish their independent contributions. Feature rankings and fitted response curves may therefore change with the inclusion of additional studies, alternative feature coding, or different model specifications. External validation was not feasible, and the ML results should consequently be regarded as exploratory and hypothesis-generating. Consistently, the internal validation results showed negative cross-validated R² values across all ML models, indicating limited out-of-sample predictive performance. Thus, the ML findings should not be used for individualized efficacy prediction or definitive dose selection. In addition, substantial residual heterogeneity persisted after subgroup analyses and exploratory meta-regression, indicating that variability among animal models, curcumin formulations, experimental protocols, sample sizes, and outcome measurements may have influenced the pooled estimates. Fifth, while HED provides a valuable translational benchmark, the intrinsic pharmacokinetic discrepancies between rodents and humans necessitate stringent dose-escalation trials in future clinical validations.

## Conclusion

In conclusion, as a pleiotropic natural compound, curcumin represents a highly promising therapeutic agent for the management of OP. Through a rigorous meta-analysis, this study has validated the robust efficacy of curcumin in mitigating bone loss across diverse preclinical models. Furthermore, the integration of ML algorithms provided an exploratory assessment of potential dose–response and time–response patterns, suggesting a preliminary model-derived candidate range around an HED of approximately 32 mg/(kg·d) and an intervention duration of 8–12 weeks. However, this ML component was constrained by the small number of study-level observations relative to the evaluated features. Given the limited number and heterogeneity of the included studies, these model-derived findings require confirmation in larger, standardized, and independently validated datasets. They should therefore be regarded as hypotheses for future translational research rather than as validated optimal therapeutic parameters or clinical dosing recommendations.
